# Shining Gold Nanostars: From Cancer Diagnostics to Photothermal Treatment and Immunotherapy

**DOI:** 10.29245/2578-3009/2018/1.1104

**Published:** 2018-01-02

**Authors:** Tuan Vo-Dinh, Yang Liu, Bridget M Crawford, Hsin-Neng Wang, Hsiangkuo Yuan, Janna K Register, Christopher G Khoury

**Affiliations:** Fitzpatrick Institute for Photonics, Department of Biomedical Engineering, Department of Chemistry, Duke University, Durham, NC 27708-0281, USA

**Keywords:** Immunotherapy, Immune checkpoint, Hyperthermia, Plasmonics, Surface-enhanced Raman scattering (SERS) Cancer

## Abstract

Cancer has been a significant threat to human health with more than eight million deaths each year in the world. There is an urgent need to develop novel methods to improve cancer management. Biocompatible gold nanostars (GNS) with tip-enhanced electromagnetic and optical properties have been developed and applied for multifunctional cancer diagnostics and therapy (theranostics). The GNS platform can be used for multiple sensing, imaging and treatment modalities, such as surface-enhanced Raman scattering, two-photon photoluminescence, magnetic resonance imaging and computed tomography as well as photothermal therapy and immunotherapy. GNS-mediated photothermal therapy combined with checkpoint immunotherapy has been found to reverse tumor-mediated immunosuppression, leading to the treatment of not only primary tumors but also cancer metastasis as well as inducing effective long-lasting immunity, i.e. an anticancer ‘vaccine’ effect.

## Introduction

Over the past few decades nanotechnology has contributed to significant advances in medicine. In particular, the use of nanoparticles has attracted great interest due to their unique efficacy and specificity in imaging, diagnostics and therapy. This article provides an overview of the research and development and applications of plasmonic nanoplatforms such as gold nanostars (GNS) developed in our laboratory for use in bioimaging, photothermal therapy (PTT) and immunotherapy. Gold nanostars provide an excellent diagnostic and therapeutic (theranostics) platform having multi-modality capability spanning surface-enhanced Raman scattering (SERS), two-photon photoluminescence imaging, photodynamic therapy, photothermal therapy, and immunotherapy. The enhanced permeability and retention feature and the capacity to efficiently convert photon energy into heat, make GNS the ideal photothermal transducer for selective cancer therapy at the nanoscale level as recently demonstrated by our group both in vitro and in vivo experiments. The use of GNS-mediated PTT in combination with immunotherapy can broaden and enhance the efficacy of immunotherapy. The combination therapy not only eradicates primary ‘treated’ tumors but also results in the immune-mediated destruction of distant ‘untreated’ metastatic tumors and creates an anticancer ‘vaccine’ effect.

## Diagnostics and Bioimaging

A special type of metallic nanoparticles, called “plasmonic” nanoparticles exhibits enhanced optical and electromagnetic properties. The term “plasmonics” is derived from the word plasmon, which refers to oscillations of conduction electrons in metallic nanostructures under the excitation light (e.g., laser) that irradiates the surface. These oscillations of electrons on the surface, called a “surface plasmon”, produce intense electromagnetic (EM) fields, leading to enormous increase in Raman scattering by 6–8 orders of magnitude. Raman spectroscopy is a sensing modality that allows for the optical detection of molecular and vibrational spectral information. This spectral “fingerprint” consists of very sharp peaks that can be easily distinguished from other targets allowing for the capability of sensing multiple targets simultaneously- or “multiplexing”. The plasmonic effect leads to a technique called surface-enhanced Raman scattering (SERS)^1^. Theoretical studies and numerical simulations have shown intense electromagnetic field enhancement in a wide variety of plasmonic structures^2–8^. The development of these plasmonic nanoplatforms has led to a wide variety of applications ranging from chemical sensing^8–10^ to biomonitoring of nucleic acid biomarkers such as DNAs, mRNAs, and microRNAs, which have demonstrated great promise as a novel class of biomarkers for early detection of various cancers^11–17^.

**Figure 1:**
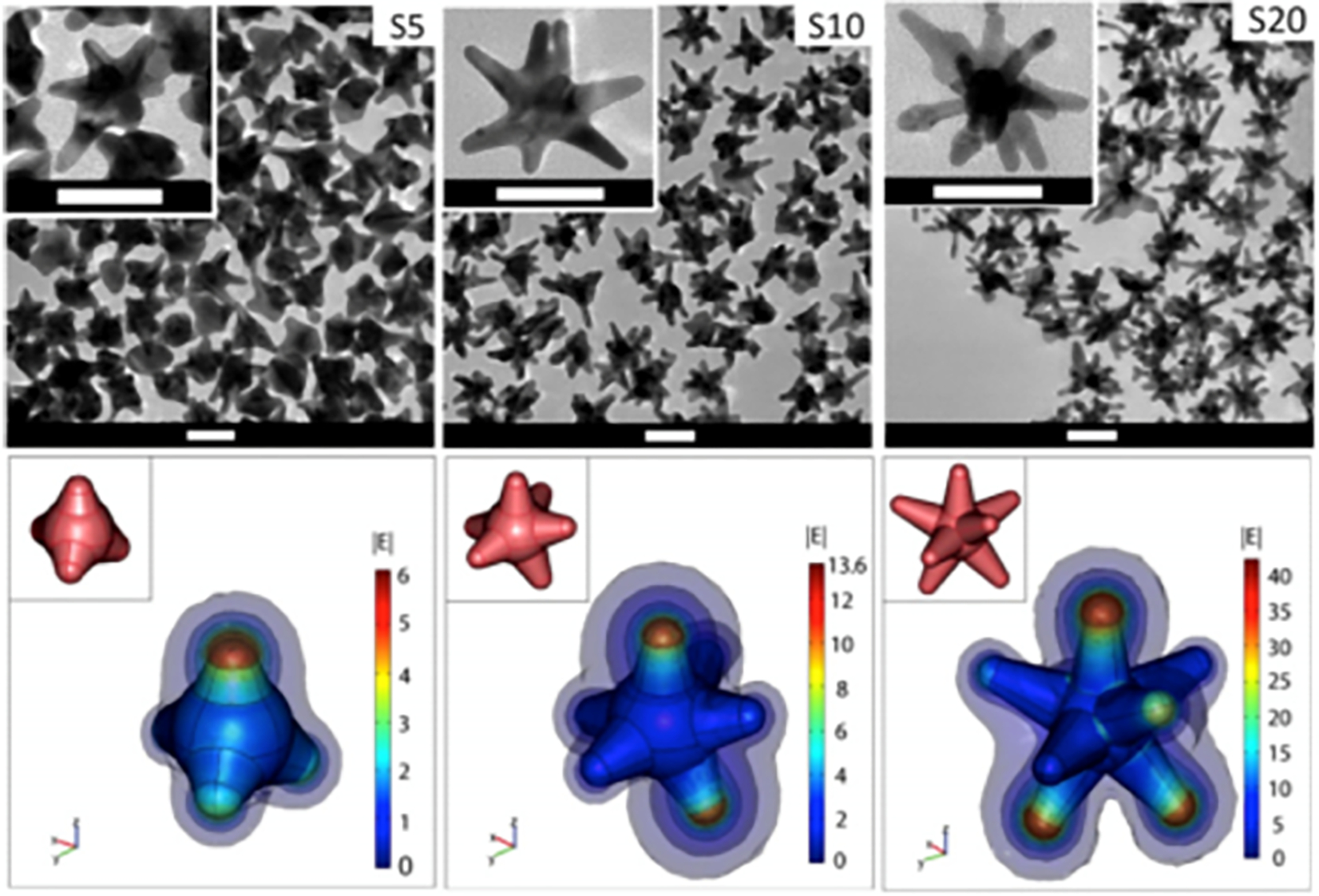
(Top) Transmission electron microscopy image of gold nanostars (GNS) having different branch numbers. Scale bar 50 nm; (Bottom) 3D modeling and simulation of |E| in the vicinity of the nanostars in response to a z-polarized plane wave incident E-field of unit amplitude, propagating in the y-direction, and with a wavelength of 800 nm. The GNS’s structure has multiple sharp branches that produce the numerous curvatures responsible for the ‘lightning rod’ effect that strongly enhances the local electromagnetic field when subject to light stimulation. The calculations indicate that the electromagnetic (EM) field at the GNS tips is dramatically enhanced, which reflects an intense tip-enhanced plasmonic effect. The insets depict the 3D geometry of the stars. Diagrams are not to scale. (Adapted from Reference 20).

Among plasmonic nanoparticles (e.g., gold nanospheres), gold nanostars (GNS) are of particular interest as they offer optical tunability by engineering subtle changes in their geometry. The multiple sharp branches on GNS create a “lightning rod” effect that enhances the local EM field dramatically (Figure. 1), leading to intense SERS signals for sensitive sensing and bioimaging. The intense EM enhancement produces strong light absorption by GNS and effective energy conversion for heating of tumor cells in nanoparticle-mediated photothermal therapy studies^19,20^. The large field enhancement at the tips of the star is due to a combination of the resonance enhancement and the “lighting-rod effect” associated with the large curvature at the tips. This curvature creates a larger surface charge density and consequently a higher electric field. As a result, nanostars can generate E-field “hot spots” that can greatly exceed the enhancement of smoother particles such as nanospheres. We have introduced a novel surfactant-free synthesis method that does not require toxic surfactant for producing biocompatible GNS suitable for in vivo applications. The synthesis method for GNS requires no addition of a toxic polymer surfactant, e.g., cetyl-trimethylammonium bromide (CTAB), rendering the GNS biocompatible and surface functionalizable. These features are important for in vivo biomedical applications^21^. In contrast to other NPs that requires hours of elaborate chemical processes, our high-yield AuNS synthesis method is simple and takes less than 30 seconds to complete. GNS optical properties can be engineered by making subtle changes in their synthesis chemistry that results in geometry changes that can tune their peak absorption band into the near infrared (NIR) spectral range (700–1200 nm)^21,22^, often referred to as the ‘diagnostic and therapeutic’ optical window, where photons travel further in tissue for sensing and therapy.

**Figure 2:**
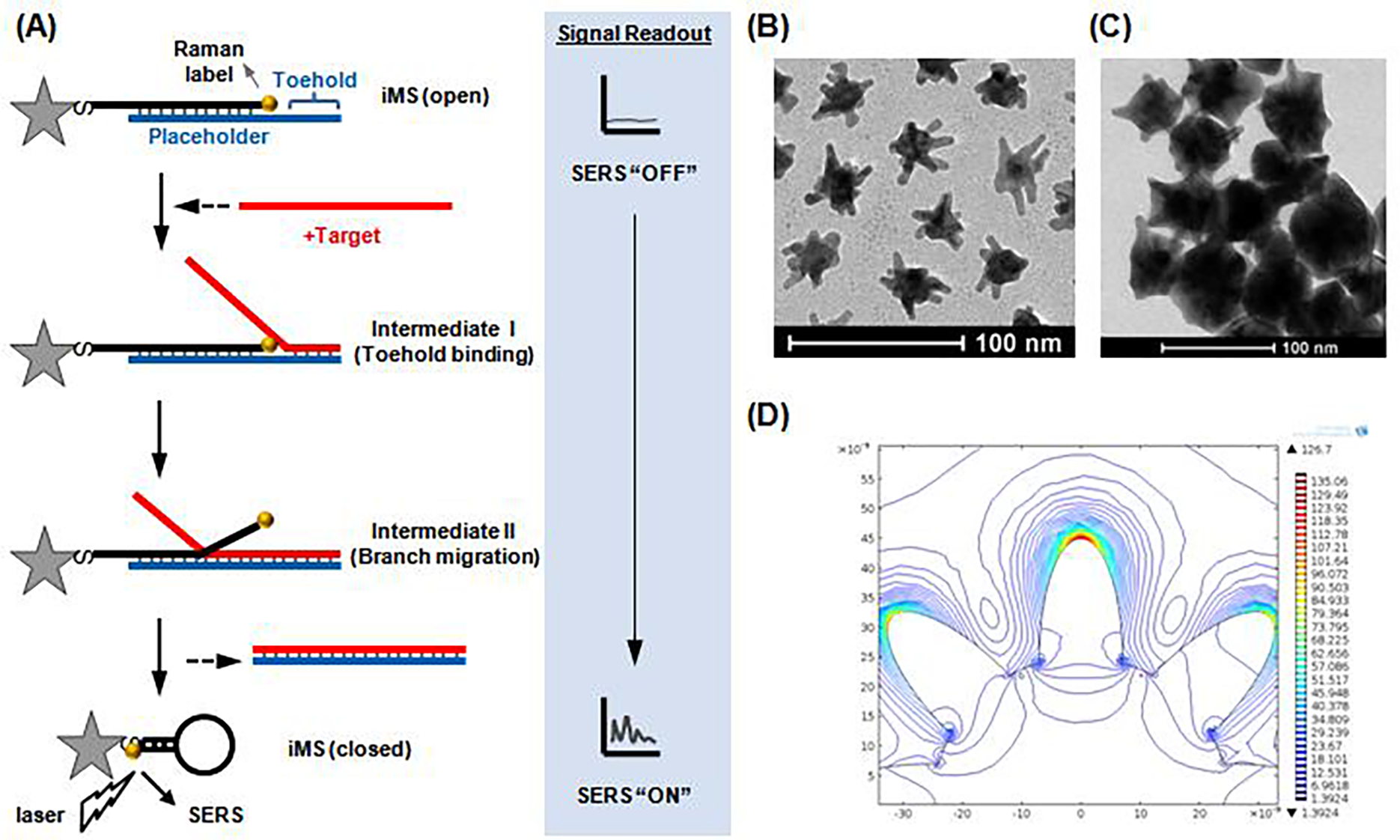
Operating principle of the “Inverse Molecular Sentinel” nanoprobe for detection of nucleic acid biomarkers of disease. Without target, the probe is “open” with low SERS signal: OFF state. Upon binding to nuclear acid target (e.g., DNA, mRNA, miRNA), the placeholder strand leaves the nanoparticle (GNS) following a strand-displacement process. The stem-loop closes, moving the Raman label onto the plasmonic nanoparticle (Adapted from Ref. 17).

We have developed a new “Off-to-On” detection scheme referred to as the “Inverse Molecular Sentinel” (iMS) nanoprobes, which combines the nucleic acid competitive strand-displacement for target recognition and binding and the plasmonic effect for sensitive SERS detection ((Figure 2.)^17^, MicroRNAs (miRNAs) have demonstrated great promise as a novel class of biomarkers for early detection of various cancers, including breast cancer. However, due to technical difficulties in detecting these small molecules, miRNAs have not been adopted into routine clinical practice for early diagnostics. Thus, it is important to develop alternative detection strategies that could offer more advantages over conventional methods. We have applied the iMS nanoprobe diagnostics technology to detect microRNA biomarkers for early cancer diagnostics^18^.

Plasmonics-enhanced and optically modulated delivery of nanostars into brain tumors in live animals was demonstrated^23,24^. The imaging method of GNS using two-photon photoluminescence (TPL), a method that combines energies of two photons in the near infrared (NIR) to induce luminescence of molecules in the visible range, provided an unprecedented spatial selectivity for enhanced targeted nanostar delivery to cortical tumor tissue. Because of the extremely high intensity of TPL emission from GNS, tracking the motion of GNS in the blood vessel can be observed in real-time^24^. A quintuple-modality nanoreporters based on GNS for SERS, TPL, magnetic resonance imaging (MRI), computed tomography (CT), and photothermal therapy (PTT) have been developed^25^. Intense TPL signals have allowed the study of GNS uptake mechanisms in vitro to track GNS in real-time and to assess the intratumor GNS distribution in vivo^23,26^.

**Figure 3.**
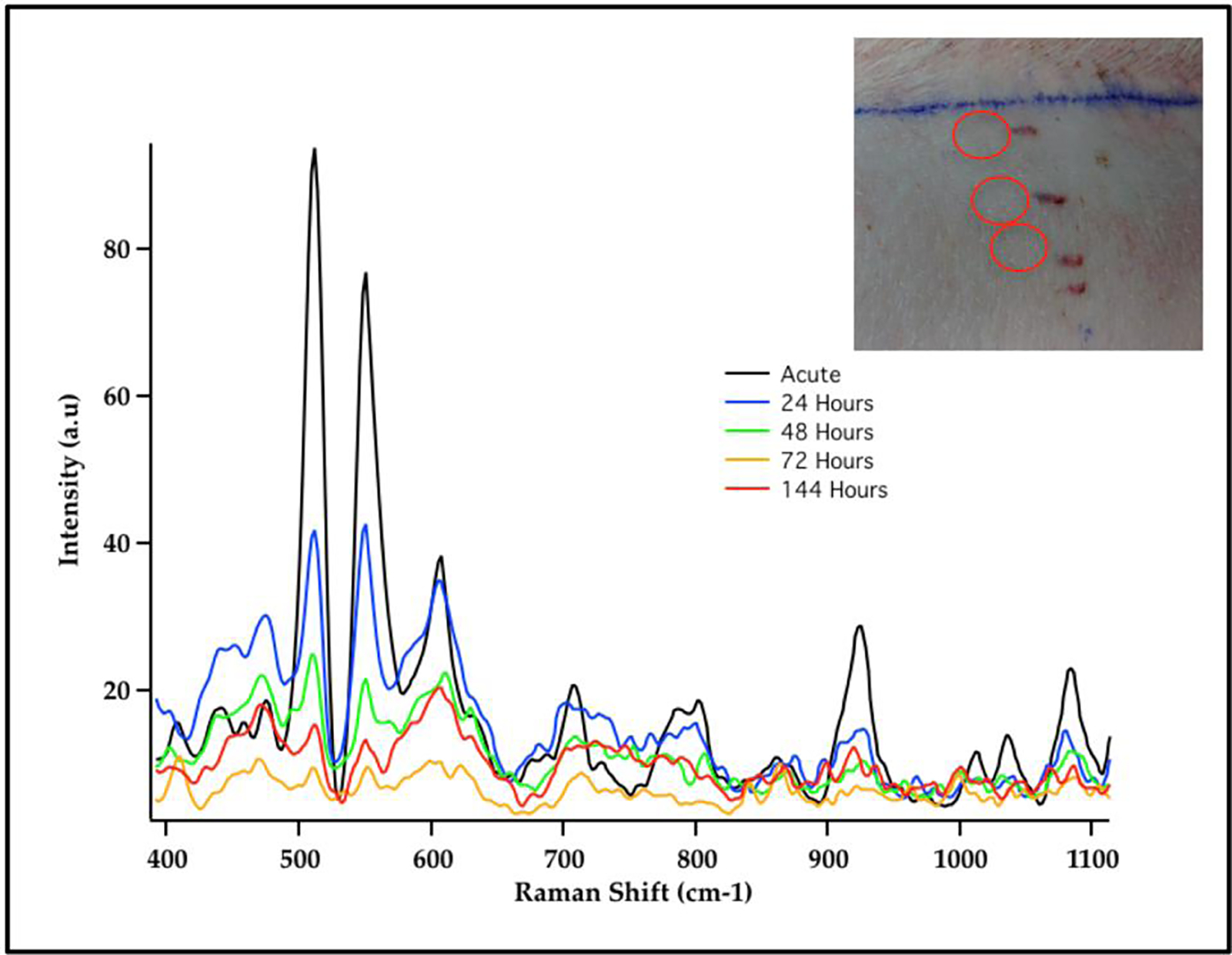
SERS Spectra of Cy7 dye-labeled gold nanostars (GNS) embedded in porous scaffold and intradermally implanted in living pig dorsum (inset). The results demonstrated the possibility to monitor in vivo nanoprobes implanted under the skin by detecting the unique SERS spectra of Cy7 dye label. [Adapted from Reference 27].

SERS measurements have also been used for sensing GNS probes in living animals^26,27^. In addition to SERS, GNS also exhibit strong TPL emission and can be used to monitor nanoparticle distribution at the cellular level without dye labeling. Furthermore GNS have been use as contrast agents for CT imaging utilizing the strong absorption characteristics of the nanoparticles to enhance the contrast of the detected x-ray image^19^. Gold nanostars that were injected intravenously into mice having sarcoma tumors exhibited intense SERS signals due to the strong local field enhancement at the tips of the nanostar spikes^19^. GNS sizes can be controlled so that they passively accumulate in tumors due to the enhanced permeability and retention (EPR) effect of tumor vasculature. The EPR effect is due to the inherent leakiness of the underdeveloped tumor vasculature, which allows nanoparticles to escape the circulation and accumulate passively in tumors. Also retention of nanoparticles in the tumor is enhanced due to the inefficient lymphatic system, which would normally carry extravasated fluid back to the circulation. Nanoparticles must be designed to have a narrow size range between approximately 10 and 100 nm to take advantage of the EPR effect. Nanoparticles larger than 100 nm are cleared by the reticuloendothelial system^28^ while those smaller than 10 nm are rapidly cleared by the kidneys^29^. GNS take advantage of the EPR effect because they can be fabricated to have hydrodynamic sizes that fit well in the 10–100 nm size ranges. The SERS signal of accumulated GNS within each tumor and contralateral leg muscle was measured. The GNS were labeled with p-mercaptobenzoic acid as the Raman reporter. The unique SERS peaks of the SERS-labeled reporter on GNS were detected in the tumor but not in the contralateral muscle, illustrating selective absorption of GNS into tumors and demonstrating the possibility of using SERS for in vivo detection and differentiating tumor from normal muscle in mouse models^19^. The SERS signal from this SERS-encoded GNS platform can be detected in human skin grafts and in large animal models (live pigs), demonstrating the clinically relevant translational capabilities of the SERS technique for in vivo biosensing (Figure 3)^27^. The use of SERS nanoprobes offers significant advantages for sensing and imaging as Raman probes do not suffer from photobleaching and photodegradation effects encountered in fluorescent probes.

## Photothermal nanotherapy

**Figure 4.**
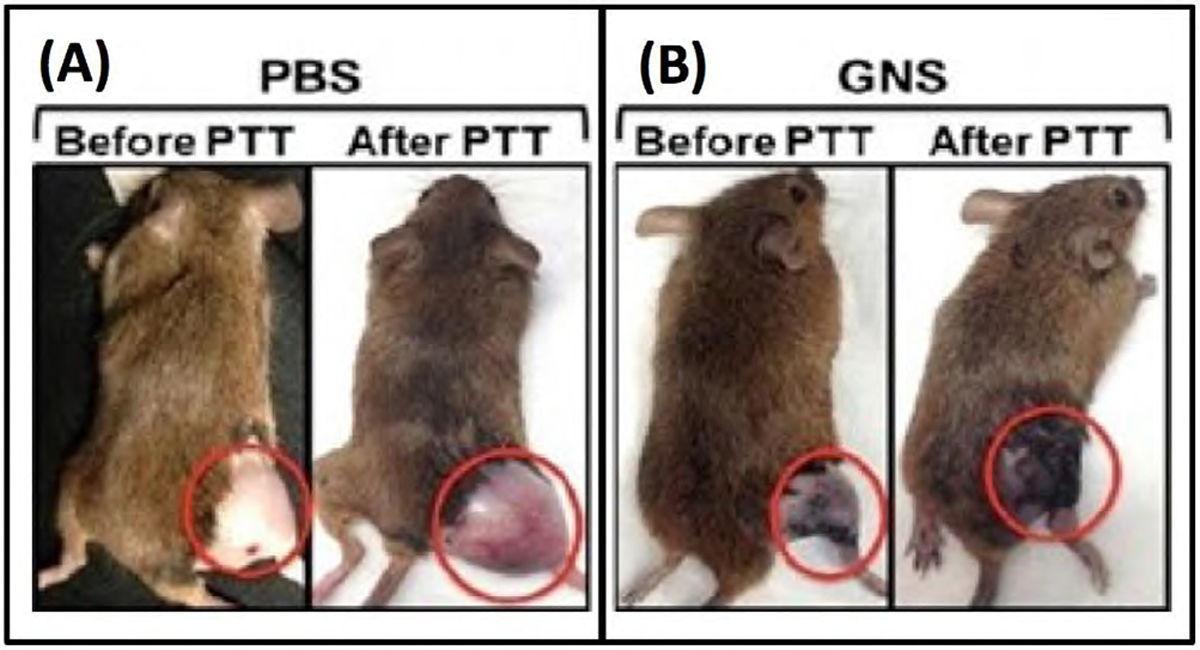
Photothermal therapy on sarcoma mice with (A) phosphate buffered saline (PBS) injection and (B) with intravenous GNS injection (B). The mice are shown before photothermlal treatment (PTT) and 3 days post PTT. [Adapted from Reference 19].

As a principle, hyperthermia (HT), which is a treatment where heat is applied to a tumor or organ, aims to increase tumor temperature above physiologic body temperature with the goal of directly inducing cellular damage, as well as promote local and systemic antitumor immune effects. Conventionally, hyperthermia is delivered using microwave, radiofrequency, high-intensity focused ultrasound, or heat applicators. However, these methods are not suitable for deep-seated tumors and the heating distribution is often not well controlled. More importantly, these methods are only macroscopically confined to the tumor area but cannot target or ablate cancer cells at the microprecision scale. Nanoparticle-mediated thermal therapy has recently received increasing interest^19,23,24,30–34^. The ability to safely target single tumor cells with a high level of efficacy and specificity can be obtained with GNS. Their multiple sharp branches acting like “lightning rods” can convert safely and efficiently light into heat. We have performed a direct measurement of photothermal conversion efficiency for the 30-nm and 60-nm GNS, and we compared their efficiency to gold nanoshells, which are one of the most well-studied nanoparticles used for photothermal therapy. The results showed the temperature profiles for each of these three nanoparticles, with the 30-nm and 60-nm GNS having a much higher equilibrium temperature than nanoshells (34.7 °C) at equivalent optical density^19^. Rapid ablation can be achieved by GNS-mediated photothermal therapy by exploting the natural propensity of nanoparticles to extravasate the tumor vascular network and accumulate in and around cancer cells^19^. The significant reduction of the laser energy needed to precisely destroy the targeted cancer cells in which GNS preferentially accumulate due to the enhanced permeation and retention (EPR) effect^35^. Figure 4 shows images of mice before and after PTT with and without injected GNS. Note, the dramatic tumor size reduction due to PTT in the mouse injected with GNS^19^. Plasmonics-enhanced and optically modulated delivery of nanostars into brain tumor in live animals has been demonstrated in a murine model^26^; photothermal treatment on tumor vasculature may induce inflammasome activation, thus increasing the permeability of the blood brain-tumor barrier. Using nanostars functionalized with cell penetrating peptides to facilitate the intracellular delivery followed by irradiation with a femto-second pulsed laser, a successful in vivo photothermal therapy was achieved under an irradiance of 0.2 W/cm^220^, which is below the maximal permissible exposure of skin per ANSI regulation. These studies demonstrated that GNS have great potential for use in photothermal cancer therapy.

## Synergistic combination immunotherapy

Immunotherapy has emerged as one of the most promising modalities to treat cancer. In recent years, immunotherapy with specific immune checkpoint inhibitor provides a promising way to break the tumor immunosuppressive environment^37,38^. Programmed death-ligand 1 (PD-L1), a protein overexpressed on cancer cell membrane, contributes to the suppression of the immune system. PD-L1 binds to its receptor, PD-1, found on activated T cells, B cells, and myeloid cells, to modulate T cell function. The therapeutic anti-PD-L1 antibody is designed to block the PD-L1/PD-1 interaction and reverse tumor-mediated immunosuppression. Blocking the PD-L1/PD-1 axis has been shown to be highly beneficial in many human tumors and used as a cancer treatment modality^39–43^. However, current antibodies work only for a limited number of patients and can become ineffective with time.

**Figure 5.**
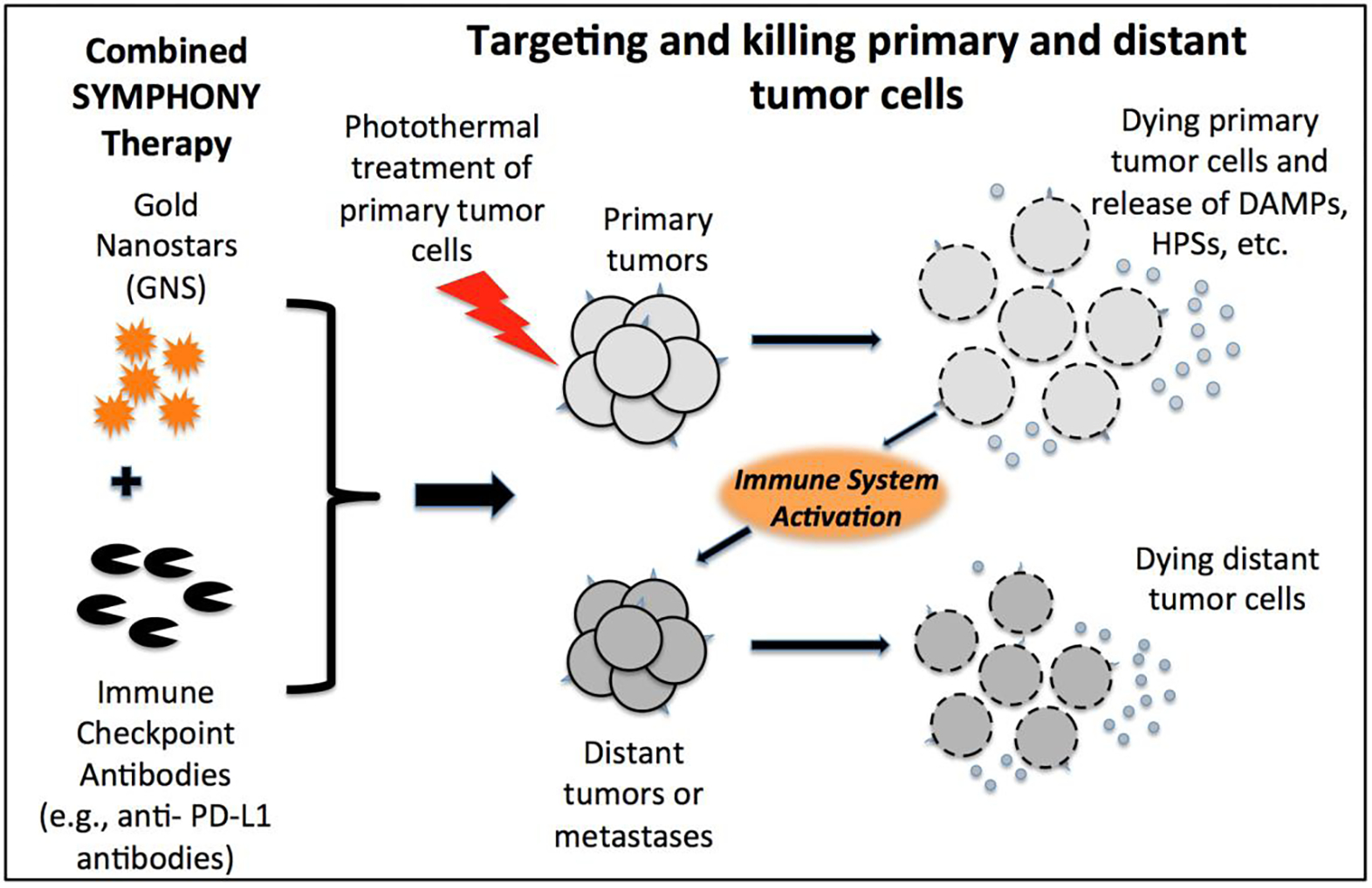
Synergistic Immuno Photo Nanotherapy (SYMPHONY) Modality to treat metastatic cancer [Adapted from Reference 44].

The combination of immune checkpoint inhibitor-based immunotherapy with GNS-mediated photothermal therapy has produced an effective two-pronged treatment modality referred to as Synergistic Immuno Photo Nanotherapy (SYMPHONY), which is designed to treat both primary and secondary tumor cells^44^. The efficacy of SYMPHONY is based on several synergistic processes (Figure. 5). First, localized PTT with GNS and NIR irradiation is used to kill primary tumor cells. Upon GNS-PTT treatment, dying tumor cells after thermal ablation could release tumor associated antigens (TAAs), damage-associated molecular pattern molecules (DAMPs), heat shock proteins (HSPs), etc. In live cells DAMPs are intracellular molecules that are normally hidden. When cells are damaged or dying, DAMPS are released and acquire immunostimulatory properties. DAMPS have been shown to exert various effects on antigen-presenting cells (APCs), such as maturation, activation and antigen processing/presentation^45^. APCs, which are present in the tissue or in local draining lymph nodes, process the tumor antigens and present tumor-derived peptides to T cells. Combining anti-PD-L1 treatment with tumor antigen presentation will activate tumor-specific T cells that will attack both the primary and distant/metastatic cancer cells. This is particularly important in the primary tumor bed, hypoxic-oxygenated boundary, where it is believed metastatic/ differentiating/proliferating potential is maximum. Mouse studies have revealed that the two-pronged therapeutic approach, combining immune-checkpoint inhibition and GNS–mediated photothermal therapy, was effective in destroying primary treated tumors as well as untreated distant tumors in mice implanted with the MB49 bladder cancer cell line^44^. The effect of the combination of plasmonic GNS-enabled photothermal ablation and PD-L1 immunomodulation was demonstrated to be synergistic and not just additive. Furthermore, the delayed rechallenge with repeated MB49 tumor injections into cured mice did not lead to new tumor formation, indicating that the combined treatment induced effective long-lasting immunity, i.e. an anticancer ‘vaccine’ effect^44,46^.

## Conclusion

Plasmonics–active gold nanostars provide an excellent multifunctional platform with capabilities spanning multi-modality sensing (SERS, TPL CT, MRI, PET) and therapy (PTT, PDT and immunotherapy). Due to their desirable features including the lack of photobleaching and photodegradation, narrow spectral fingerprints, and their capability to allow multiplexed bioanalysis, GNS will open new opportunities to important theranostics applications. GNS have a tunable plasmonic band in the near infrared region, where there is low tissue absorption and autofluorescence, and therefore they are quite suitable for in vivo applications. Furthermore, the combination of checkpoint blockade immunotherapy with GNS-mediated photothermal therapy offers the promise to address one of the most challenging problems in the treatment of metastatic cancer and ultimately producing an effective treatment capable of inducing effective long-lasting immunity against cancer.

## Future Directions

Gold nanostars is a versatile platform showing great promise for cancer management. The following directions could be further pursued to improve cancer imaging and treatment using GNS. In addition to passive targeting via the EPR effect, future studies can investigate active targeting using peptides or antibodies linked to GNS in order to improve tumor uptake. Of special interest is the synergistic combinatorial approach such as SYMPHONY that can reverse tumor-mediated immunosuppression, showing promise to treat not only unresectable primary tumors, but also distant cancer metastasis by enhancing the systemic activity of specific and adaptive immune responses and inducing an anticancer “vaccine” effect. Further studies will provide better understanding of the mechanisms underlying the novel synergistic treatment modalities of SYMPHONY in order to enhance and broaden the effect of immune-checkpoint inhibitors for successful eradication of metastatic cancer. Identifying, characterizing and investigating the specific immune cells and molecular processes involved in this synergistic interaction will pave the way for successful treatment of locally advanced and metastatic cancer, and recurring tumors.

## Abbreviation List

ANSI: American National Standards Institute
APCs: antigen-presenting cells
CT: computed tomography
DAMPs: damage-associated molecular pattern molecules
EM: electromagnetic
EPR: enhanced permeability and retention
GNS: gold nanostars
HSPs: heat shock proteins
HT: hyperthermia
MRI: magnetic resonance imaging
NIR: near infrared (NIR)
PD-L1: programmed death-ligand 1
PTT: photothermal therapy
ROS: reactive oxygen species
SERS: surface-enhanced Raman scattering
SYMPHONY: Synergistic Immuno Photothermal Nanotherapy
TAA: tumor-associated antigen
TPL: two-photon photoluminescence
